# Transcriptome profiling of mouse brain and lung under Dip2a regulation using RNA-sequencing

**DOI:** 10.1371/journal.pone.0213702

**Published:** 2019-07-10

**Authors:** Rajiv Kumar Sah, Analn Yang, Fatoumata Binta Bah, Salah Adlat, Ameer Ali Bohio, Zin Mar Oo, Chenhao Wang, May Zun Zaw Myint, Noor Bahadar, Luqing Zhang, Xuechao Feng, Yaowu Zheng

**Affiliations:** 1 Transgenic Research Center, School of Life Sciences, Northeast Normal University, Changchun, China; 2 Key Laboratory of Molecular Epigenetics of Ministry of Education, Northeast Normal University, Changchun, China; Southern Methodist University, UNITED STATES

## Abstract

Disconnected interacting protein 2 homolog A (DIP2A) is highly expressed in nervous system and respiratory system of developing embryos. However, genes regulated by *Dip2a* in developing brain and lung have not been systematically studied. Transcriptome of brain and lung in embryonic 19.5 day (E19.5) were compared between wild type and *Dip2a*^-/-^ mice. An average of 50 million reads per sample was mapped to the reference sequence. A total of 214 DEGs were detected in brain (82 up and 132 down) and 1900 DEGs in lung (1259 up and 641 down). GO enrichment analysis indicated that DEGs in both Brain and Lung were mainly enriched in biological processes ‘DNA-templated transcription and Transcription from RNA polymerase II promoter’, ‘multicellular organism development’, ‘cell differentiation’ and ‘apoptotic process’. In addition, COG classification showed that both were mostly involved in ‘Replication, Recombination, and Repair’, ‘Signal transduction and mechanism’, ‘Translation, Ribosomal structure and Biogenesis’ and ‘Transcription’. KEGG enrichment analysis showed that brain was mainly enriched in ‘Thyroid cancer’ pathway whereas lung in ‘Complement and Coagulation Cascades’ pathway. Transcription factor (TF) annotation analysis identified Zinc finger domain containing (ZF) proteins were mostly regulated in lung and brain. Interestingly, study identified genes *Skor2*, *Gpr3711*, *Runx1*, *Erbb3*, *Frmd7*, *Fut10*, *Sox11*, *Hapln1*, *Tfap2c* and *Plxnb3* from brain that play important roles in neuronal cell maturation, differentiation, and survival; genes *Hoxa5*, *Eya1*, *Errfi1*, *Sox11*, *Shh*, *Igf1*, *Ccbe1*, *Crh*, *Fgf9*, *Lama5*, *Pdgfra*, *Ptn*, *Rbp4* and *Wnt7a* from lung are important in lung development. Expression levels of the candidate genes were validated by qRT-PCR. Genome wide transcriptional analysis using wild type and *Dip2a* knockout mice in brain and lung at embryonic day 19.5 (E19.5) provided a genetic basis of molecular function of these genes.

## Introduction

DIP2A is a member of disconnected (disco)-interacting 2 (DIP2) protein family whose molecular anatomical function remains to be clarified. *Dip2a* was firstly identified in Drosophila as a novel transcription factor that interacts with disconnected (disco) gene needed for proper neural connection during visual system development in Drosophila [1–3]. Previous studies have shown that *Dip2a* is highly expressed in human brain and may play a role in axon patterning in Central Nervous System (CNS) [4]. Bioinformatics analysis using Homologene suggests that DIP2A is a receptor molecule with DMAP, AMP and CAIC binding domains [5]. At DNA replication site, DIP2A, in a complex with DNA methyltransferase 1-associated protein 1 (DMAP1)—DNA (cytosine-5) -methyltransferase 1 (DNMT1)—Histone deacetylases (HDAC), regulates neurite outgrowth and synaptic plasticity [6]. Moreover, *Dip2a* has been previously identified as a risk gene associated with neurodevelopment diseases like autism spectrum disorder, development dyslexia and Alzheimer diseases [7–9]. All of these evidences strongly support the role of *Dip2a* gene in both vertebrate and invertebrate nervous system development. However, which biological process or molecular function is regulated by *Dip2a* gene during embryonic brain development is not known.

Earlier, using *Dip2a*^-/-^-LacZ knockin mice [10], we notice that *Dip2a* is highly expressed in brain neurons, retinal ganglion cell, reproductive, vascular and Lung tissue in adult and ectodermal tissue in developing embryos. RNA sequencing (RNA-Seq) has rapidly emerged as a favorite approach for high throughput gene expression and function studies. Through RNA-Seq, gene expression and gene interactions at any time point or in a particular tissue can be investigated [11]. In present study, Transcriptome (RNA-seq) analysis of E19.5 brain and lung of WT and *Dip2a*^-/-^ embryo was performed.

*Dip2a* role in brain and lung development has not been studied before. A global Transcriptome analysis of brain and lung will help us in understanding of *Dip2a* function in regulating brain and lung development. A total of 214 genes in brain and 1900 genes in lung were identified differentially expressed under *Dip2a*, suggesting that these genes are potentially relevant to brain and lung development and function. Those genes are further explicated and discussed in this study.

## Materials and methods

### Animals

*Dip2a* specific knockout transgenic mice (*Dip2a*^*-/-*^) was generated in the lab using CRISPR-Cas9 technology as previously described [12]. All mice were genotyped by PCR from tail DNA. All procedures were conducted following guidelines recommended in the guide for Care and Use of Laboratory Animals of National Institutes of Health with approval of Institutional Animal Care and Use Committee of Northeast Normal University (NENU/IACUC, AP2013011). Mice were housed in clean facility in individual IVC cages under a normal 12:12h light:dark cycles in a temperature of 20°C and humidity 50 ± 20% in Northeast Normal University. All mice were anesthetized before euthanasia with 1% pentobarbital at a dose of 10mg/kg and all effort was made to minimize suffering.

### RNA isolation and library preparation for RNA-Seq

Total RNA from brain and lung of E19.5 *Dip2a*^-/-^ and wild type embryos was isolated by using RNAiso plus reagent (Takara, Dalian) in accordance with the manufacturer’s instruction and followed by additional step of DNase I digestion to eliminate genomic DNA contamination. The quality and purity of RNA was checked by Nano drop ND-1000 spectrophotometer (Thermo Fisher Scientific, USA) and Agilent 2100 Bio analyzer (Santa Clara, CA, USA).

A total amount of 1 μg RNA per sample was used as input material for the RNA sample preparations. Sequencing libraries were generated using NEBNext UltraTM RNA Library Prep Kit for Illumina (NEB, USA) following manufacturer's recommendations and index codes were added to attribute sequences to each sample. The clustering of the index-coded samples was performed on a cBot Cluster Generation System using TruSeq PE Cluster Kit v4-cBot-HS (Illumina) according to the manufacturer's instructions. After cluster generation, the library preparations were sequenced on an Illumina Hiseq^™^ 2500 platform (Biomarker, Beijing, China) and paired-end reads were generated.

### Sequence Mapping, assembly and gene functional annotation

Raw data (raw reads) of fastaq format were firstly processed through in-house Perl scripts. In this step, clean data (clean reads) were obtained by removing reads containing adapter, reads containing poly-N and low quality reads from raw data. At the same time, Q20, Q30, GC-content and sequence duplication level of the clean data were also calculated. The clean reads were then mapped to mouse reference genome using Bowtie2 and Tophat 2 that allows up to two mismatches. Reads were assembled into transcript with Cufflink. Isolated and annotated based on the reference genome. The mRNA-Seq raw data are available at the Sequence Read Archive (https://www.ncbi.nlm.nih.gov/bioproject/PRJNA540099/) under the accession number PRJNA540099. Gene function of the mapped reads (unique transcripts) was annotated based upon the following databases: Nr (NCBI non-redundant protein sequences); Nt (NCBI non-redundant nucleotide sequences); KOG/COG (Clusters of Orthologous Groups of proteins); EggNOG; KO (KEGG Ortholog database) and GO (Gene Ontology).

### Gene expression quantification and analysis of differentially expressed genes (DEG)

Quantification of transcript expression levels was presented by FPKM (fragments per kilo base of exon per million fragments mapped) that minimize the reads output variations between samples. In order to identify DEGs between WT and Dip2a^-/-^ embryos in brain and lungs, we used DESeq software from R package. Resulting P values were adjusted using the Benjamini and Hochberg's approach for controlling the false discovery rate (FDR). DEGs with a threshold FDR adjusted, p value<0.001 and fold change ≥ 2 (log2> ±1) were selected for further analysis. Gene Ontology (GO) enrichment analysis of DEGs was implemented by GOseq R packages. KOBAS was used to test the statistical enrichment of DEGs in KEGG pathways. For transcription factor analysis, Genes were subjected under Animal TFDB database (Zhang et al., 2012).

### Quantitative real time PCR (qPCR) validation of RNAseq

One microgram of total RNA from brain and lung tissue of E19.5 WT and Dip2a^-/-^ embryos was reverse transcribed using primescriptTM^II^ cDNA synthesis kit (Takara, Dalian, China). QPCR was performed using Thermo cycler (Analytik Jena AG, Jena, Germany) and SYBR II premix (Takara, Dalian, China). All results were normalized to housekeeping gene 18S ribosomal RNA and relative quantification was calculated using comparative threshold cycle (2^-ΔΔCt^) values for 3 biological replicates.

## Results and discussion

### Gene expression profiling of brain and lung from WT and Dip2a^-/-^ mice

**Fig 1:**
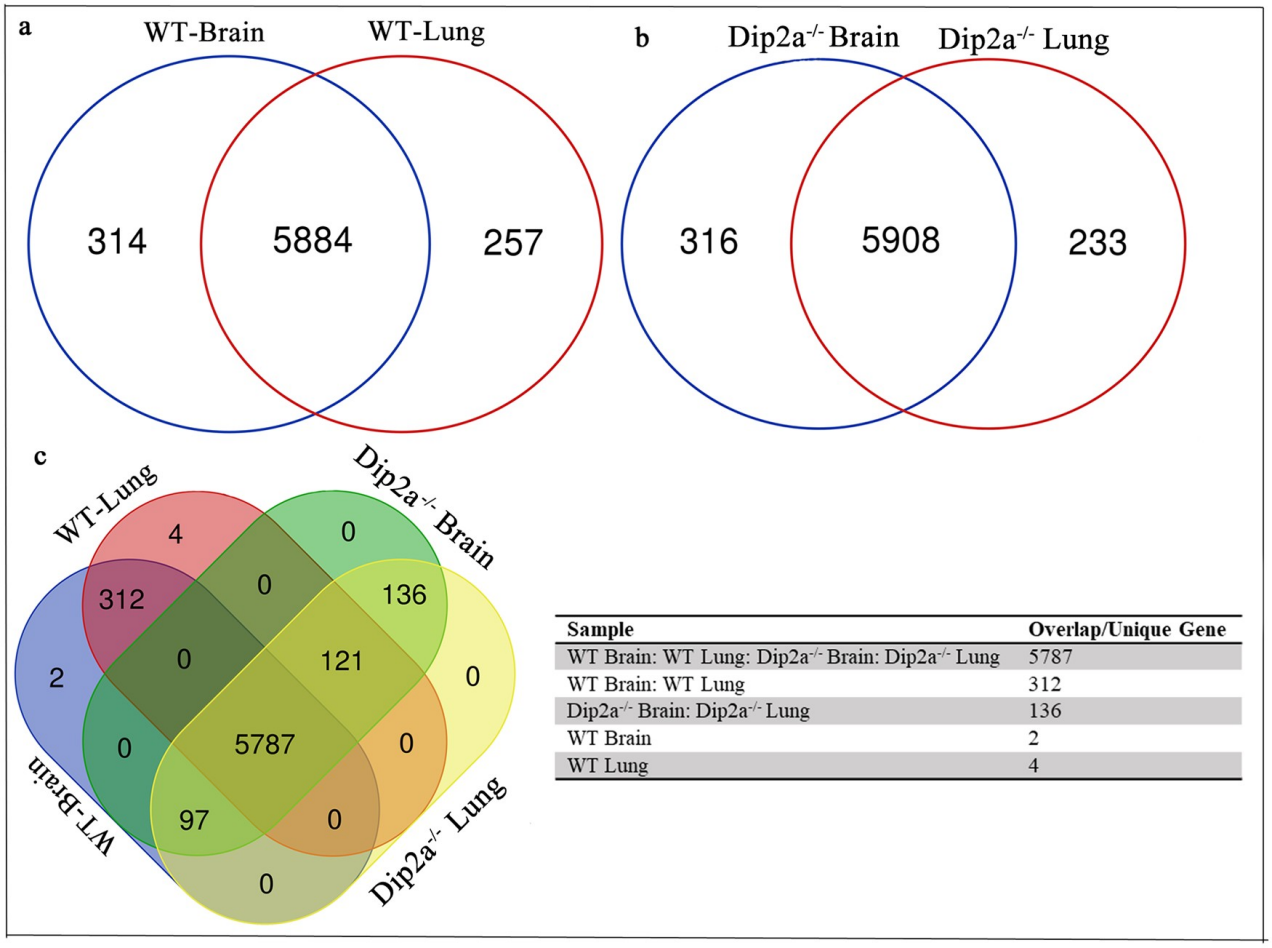
Venn diagrams showing overlap and unique unigenes identified within Wild type (WT) brain, Wild type (WT) lung, *Dip2a*^-/-^ brain and *Dip2a*^-/-^ lung. (a) In WT group, total 5884 genes were expressed in both samples, 314 genes unique to brain and 257 genes unique to lung (b) In *Dip2a*^-/-^ group, total 5908 genes were expressed in both samples, 319genes were unique to brain and 233 unigenes were unique to lung. (C) 5787 genes were expression in all samples, 2 genes were unique to WT brain and 4 genes were unique to WT lung.

Four cDNA libraries were prepared from brain and lung of WT and *Dip2a*^-/-^ E19.5 embryos (n = 3; biological replicates per sample) and sequenced using Illumina Hiseq^™^ 2500. After filtering out adaptors sequence and low quality reads, 24.08 GB of Clean Data were obtained, or 6.02 GB per sample, with a Q30 base percentage above 92.49%. The clean reads from each sample were then mapped to mouse reference genome (ftp://ftp.ensembl.org/pub/release-78/fasta/mus_musculus/) and quantification of transcripts expression levels were calculated and presented by FPKM. As shown in Table 1, the matching efficiency between the clean read and the reference genome of each sample ranged from 89.25% to 91.95%. On an average, about 6000 genes were expressed in each sample. Genes comparison between WT and *Dip2a*^-/-^ identified 5787 genes overlap in all sample and only 2 and 4 genes were unique in WT brain and WT lung respectively (Fig 1).

**Table 1:** Statistics of sequence output.

Sample	Total clean reads	Total mapped reads	Q30%	GC %
**WT brain**	40,288,890	37,047,078 (91.95%)	96.99	46.12
**WT lung**	54,035,446	48,953,863 (90.60%)	96.87	47.35
***Dip2a***^**-/-**^ **brain**	52,633,352	47,472,439 (90.19%)	96.78	46.89
***Dip2a***^**-/-**^ **lung**	54,634,002	48,763,438 (89.25%)	96.8	47.97

### Identification of differentially expressed genes and functional annotation

**Fig 2:**
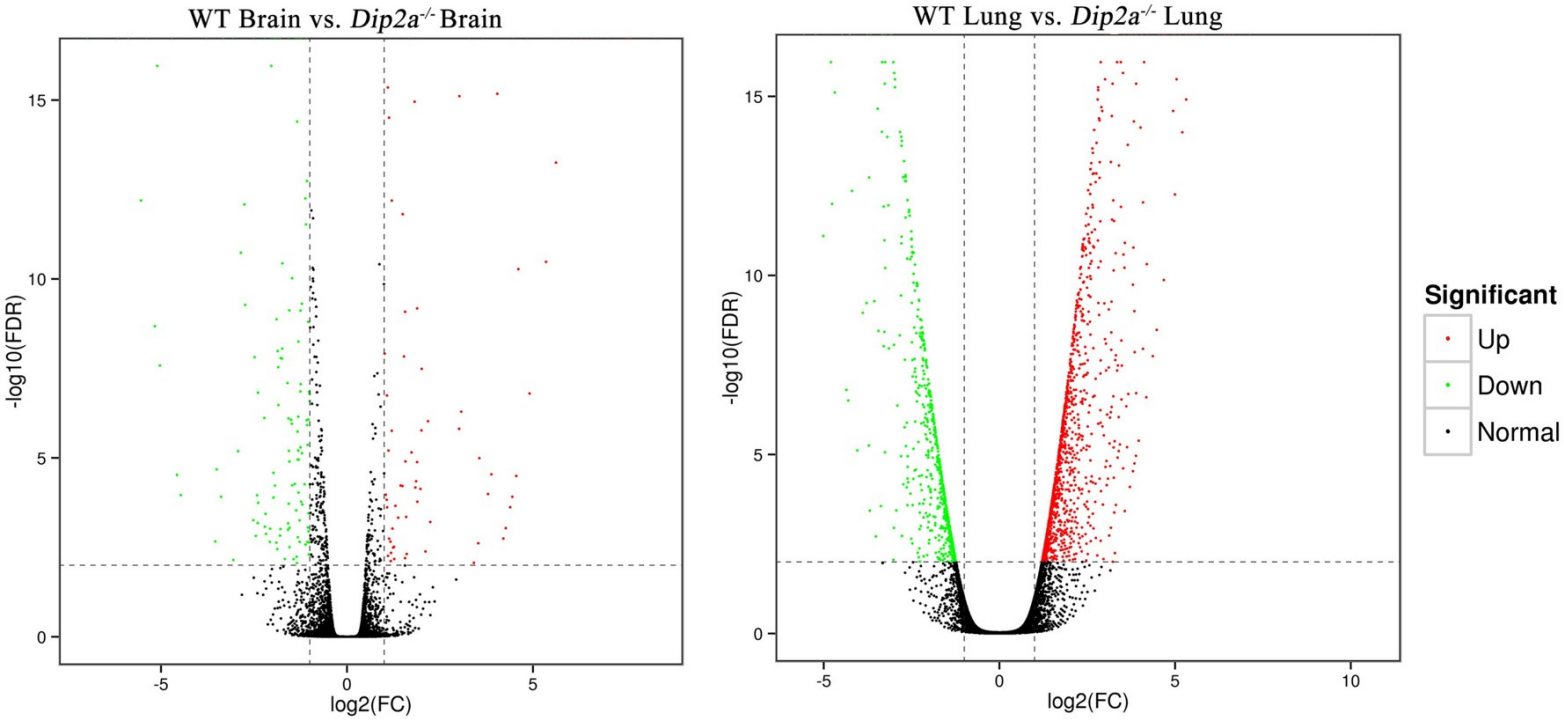
Differentially expressed genes volcano map. (a) WT brain vs. *Dip2a*^-/-^ brain. (b) WT lung vs. *Dip2a*^-/-^ lung. The red and green dots in the figure represent up-regulated and down-regulated differentially expressed genes respectively.

To identify differentially expressed genes, unigenes from WT brain vs. *Dip2a*^-/-^ brain and WT lung vs. *Dip2a*^-/-^ lung were compared. DESeq identified 214 genes in brain and 1900 genes in lung to be differentially expressed, with Fold Change ≥2 and FDR < 0.01. In *Dip2a*^-/-^ brain, 82 genes were up-regulated and 132 genes were down-regulated whereas in *Dip2a*^-/-^ lung, 1259 genes were up-regulated and 641 genes were down-regulated when compared to WT (Fig 2). In *Dip2a*^-/-^ brain, *Rpsa-ps10*, *Tpm3-rs7*, *Amd2* and *Gm8730*, *Gm10709*, *Gm6768* and *Gm9825* genes were highly over expressed whereas *Acp5*, *Ifi204*, *Col10a1*, *Ibsp* and *Mmp13* genes were highly under expressed. Similarly, in *Dip2a*^-/-^ lung, genes like *Rps2-ps6*, *Gm10709*, *Bhmt* and *Gm8730* were highly increased whereas genes like *Il1r2*, *Nr4a3*, *Cela1* and *Dlk2* were significantly decreased (Table 2). Functional annotation of brain and lung DEGs shows that more than 90% of DEGs from brain and lung had significant matches in Nr, EggNOG, GO, COG, KEGG and Swiss-Prot database respectively (S1 Table).

**Table 2:** Highly significant differentially expressed genes in *Dip2a*^-/-^ group compared to Wild type group (FDR< 0.01, FC> 20).

**Gene ID**	**Gene symbol**	**WT Brain** **FPKM**	***Dip2a*^*-/-*^ Brain** **FPKM**	**FDR**	**log2FC**
ENSMUSG00000047676	*Rpsa-ps10*	0.91	294.08	0	8.28
ENSMUSG00000058126	*Tpm3-rs7*	0.20	44.44	0	7.66
ENSMUSG00000063953	*Amd2*	0	5.41	0	7.60
ENSMUSG00000063696	*Gm8730*	0.07	22.26	0	7.51
ENSMUSG00000074516	*Gm10709*	0.66	90.26	0	7.01
ENSMUSG00000021908	*Gm6768*	0.02	4.32	0	6.70
ENSMUSG00000096403	*Gm9825*	0.57	55.52	0	6.59
ENSMUSG00000001348	*Acp5*	4.33	0.15	0	-4.70
ENSMUSG00000073489	*Ifi204*	0.97	0	2.63E-08	-5.02
ENSMUSG00000029307	*Dmp1*	1.54	0.039	1.11E-16	-5.09
ENSMUSG00000039462	*Col10a1*	0.75	0	2.10E-09	-5.16
ENSMUSG00000029306	*Ibsp*	5.75	0.13	0	-5.38
ENSMUSG00000050578	*Mmp13*	3.90	0.04	0	-6.16
**Gene ID**	**Gene Name**	**WT Lung** **FPKM**	***Dip2a***^***-/-***^ **Lung** **FPKM**	**FDR**	**log2FC**
ENSMUSG00000096403	*Gm9825*	0.005	80.34	0	10.62
ENSMUSG00000095427	*Rps2-ps6*	0.17	30.4	0	7.12
ENSMUSG00000074516	*Gm10709*	0.25	34.49	0	6.65
ENSMUSG00000074768	*Bhmt*	2.9	242.2	0	6.53
ENSMUSG00000063696	*Gm8730*	1.7	148.4	0	6.41
ENSMUSG00000047676	*Rpsa-ps10*	2.63	144.52	0	5.83
ENSMUSG00000032315	*Cyp1a1*	0.28	13.25	0	5.55
ENSMUSG00000045027	*Prss22*	0.55	23.05	0	5.32
ENSMUSG00000078956	*Gm14221*	5.32	1.12	1.01E-14	5.2
ENSMUSG00000026073	*Il1r2*	14.53	0.64	0	-4.27
ENSMUSG00000028341	*Nr4a3*	24.47	0.99	0	-4.48
ENSMUSG00000023031	*Cela1*	10.47	0.23	7.77E-16	-4.68
ENSMUSG00000047428	*Dlk2*	3.84	0.086	1.01E-12	-4.76
ENSMUSG00000049796	*Crh*	6.74	0.13	1.11E-16	-4.79
ENSMUSG00000027313	*Chac1*	10.42	0.2	0	-5.23
ENSMUSG00000020591	*Ntsr2*	10.57	0.08	0	-5.56

### GO enrichment analysis and COG classification of Dip2a-regulated DEGs

**Fig 3:**
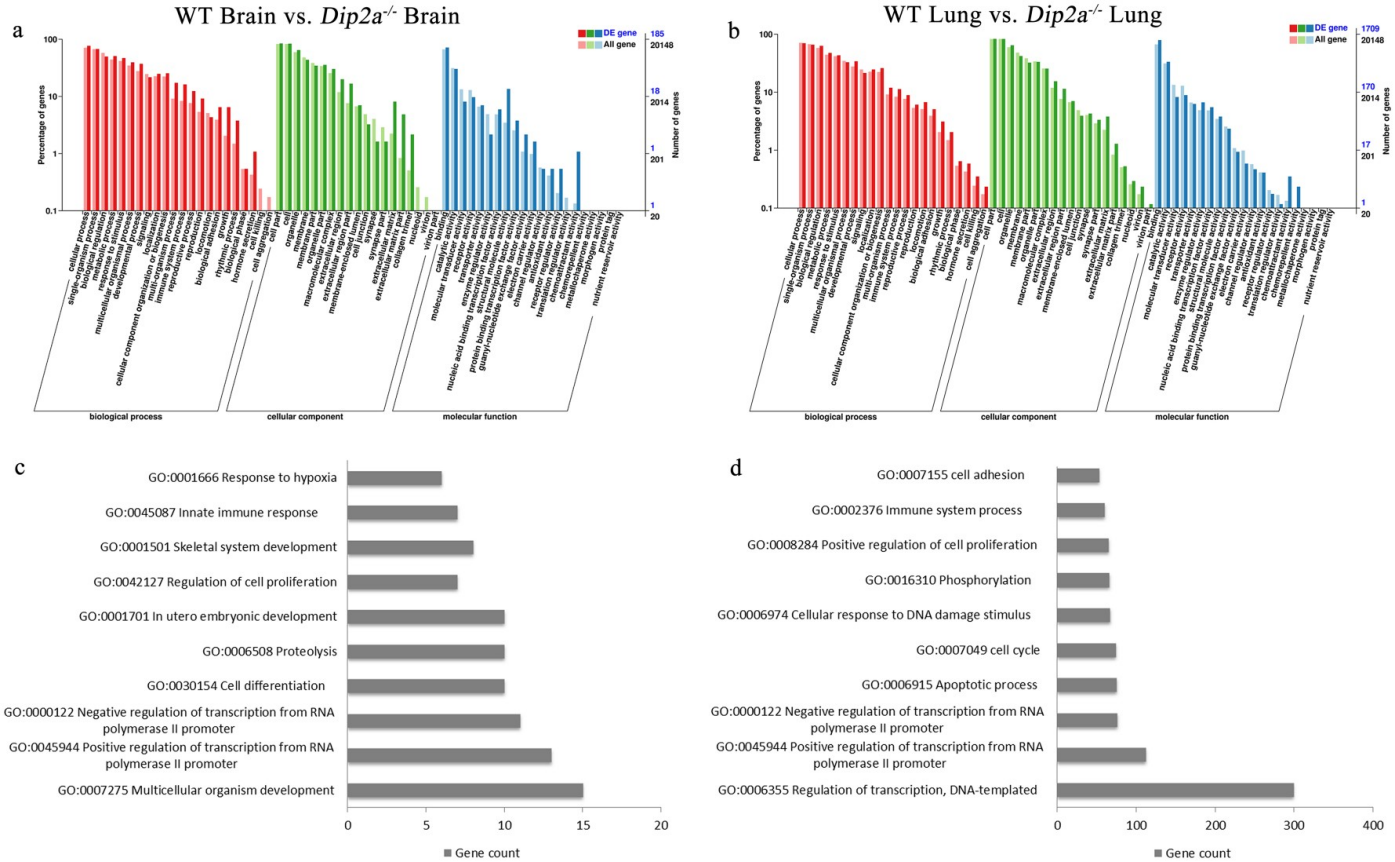
Gene Ontology (GO) classification of DEGs from WT brain vs. *Dip2a*^-/-^ brain (a, c) and WT lung vs. *Dip2a*^-/-^ lung (b, d). (a,b) Histogram of GO annotation was generated by KOBAS (kobas.cbi.pku.edu.cn). The X-axis indicates GO classification, the Y-axis on the left indicates the percentage of genes, and the Y-axis on right indicates the number of genes. One gene could be assigned with more than one GO term. (c,d) Most significant enriched biological terms in brain and lung.

**Fig 4:**
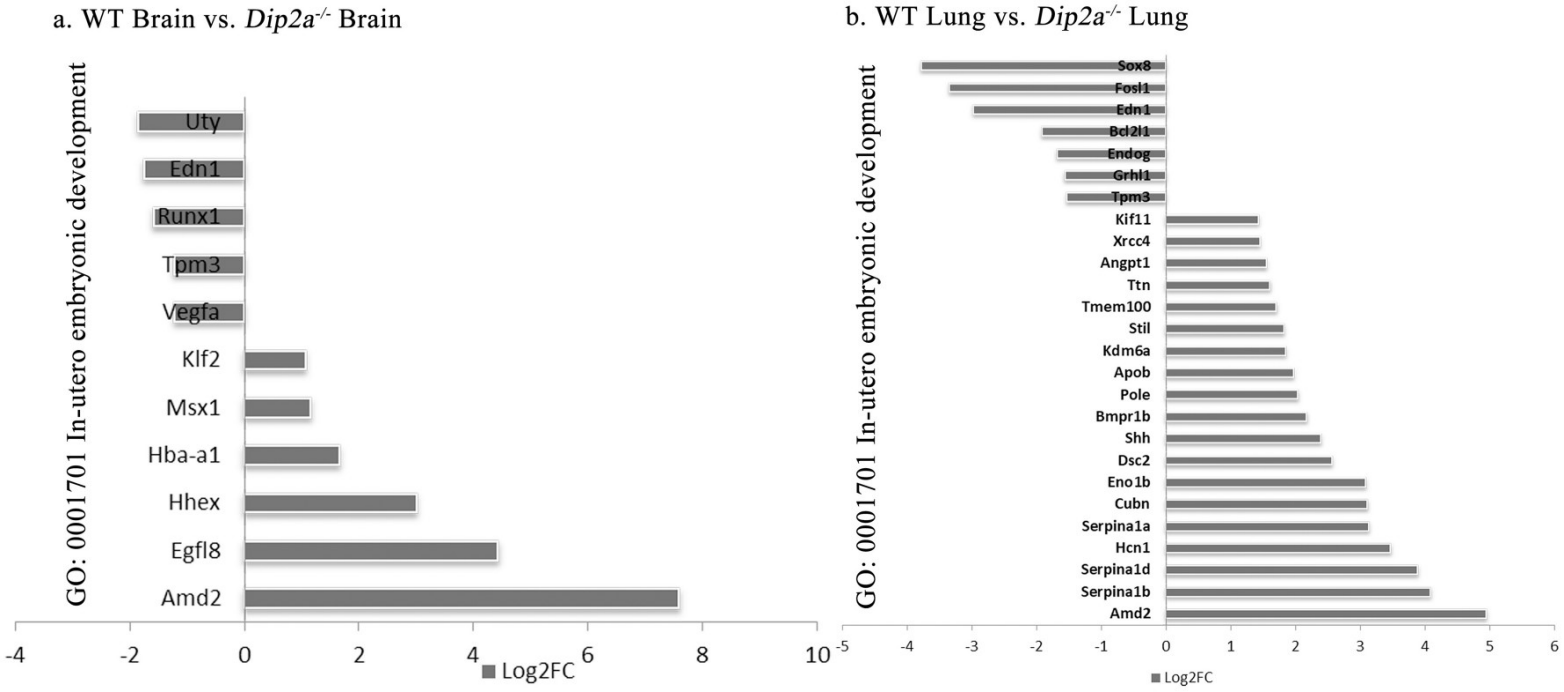
List of DEGs annotated to GO term ‘*In-utero* embryonic development’.

For gene ontology (GO) analysis, 185 DEGs from brain and 1709 DEGs from lung were classified into three GO categories and 51 terms (Fig 3). In biological process category, most of the DEGs in brain and lung were assigned to ‘cellular process’, ‘single-organism process’ and ‘metabolic process’. In molecular function category, most DEGs were annotated under ‘binding’, ‘catalytic activity’ and ‘signal transducer activity’. Within cellular component, ‘cell’, ‘cell part’ and ‘organelle’ was annotated with most DEGs. To further clarify the biological process, DEGs from both groups were enriched in 84 terms and the 10 most significant terms from each groups are summarized in Fig 3(c) and 3(d). In lung, the most significant biological terms include ‘regulation of transcription, DNA-templated’ and ‘positive-negative regulation of Transcription from RNA polymerase II promoter’ and ‘apoptotic process’. In brain, the most significant terms were ‘multicellular organism development’, ‘positive-negative regulation of Transcription from RNA polymerase II promoter’ and ‘cell differentiation’. In addition, 34 DEGs from lung and 12 DEGs from brain were annotated under GO term ‘in utero embryonic development’ (Fig 4). These DEGs are important in progression of embryo in uterus over time.

**Fig 5:**
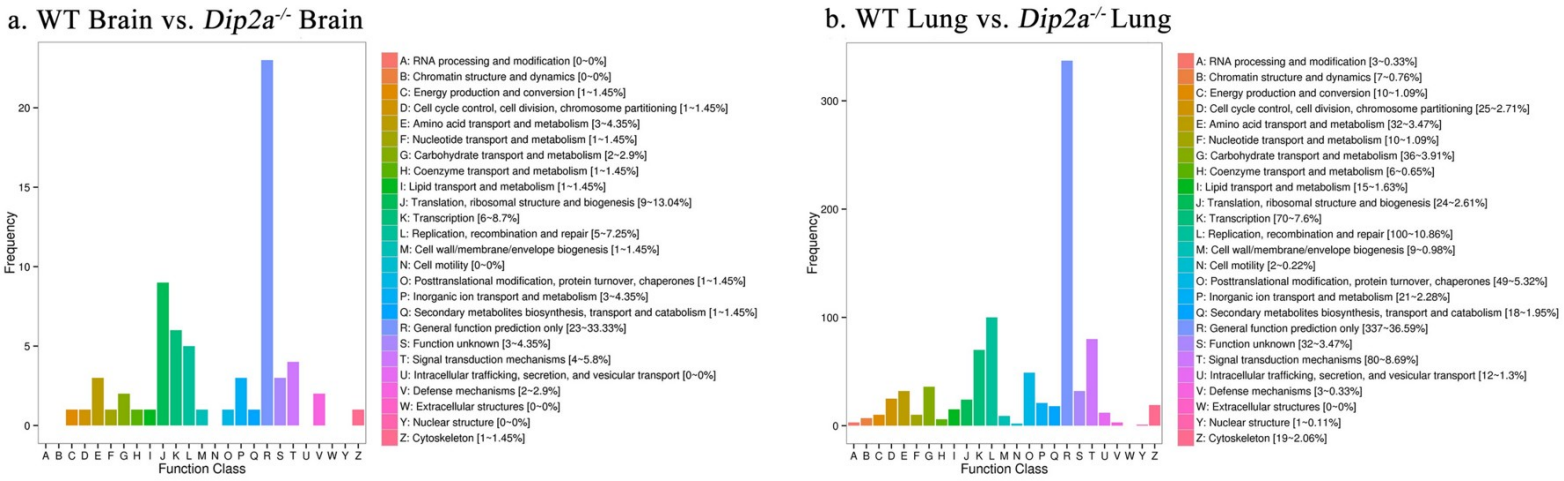
COG classification of differential expression genes (a) WT brain vs. Dip2a^-/-^ brain and (b) WT lung vs. Dip2a^-/-^ lung. The X-axis indicates content of each category of COG and the Y-axis indicates number of genes annotated in each category.

To further clarify the molecular function of *Dip2a*, total 54 and 677 DEGs from brain and lung were assigned to COG classification and divided into 26 specific categories (Fig 5). In both groups, the top hits include ‘Replication, Recombination and repair (7.25% & 10.86%)’, ‘Signal transduction and mechanism (5.8% and 8.69%)’, ‘Translation, Ribosomal structure and Biogenesis (2.61% &13.04%)’ and ‘Transcription (7.6% & 8.7%)’.

### KEGG pathway annotation of brain and lung DEGs

**Fig 6:**
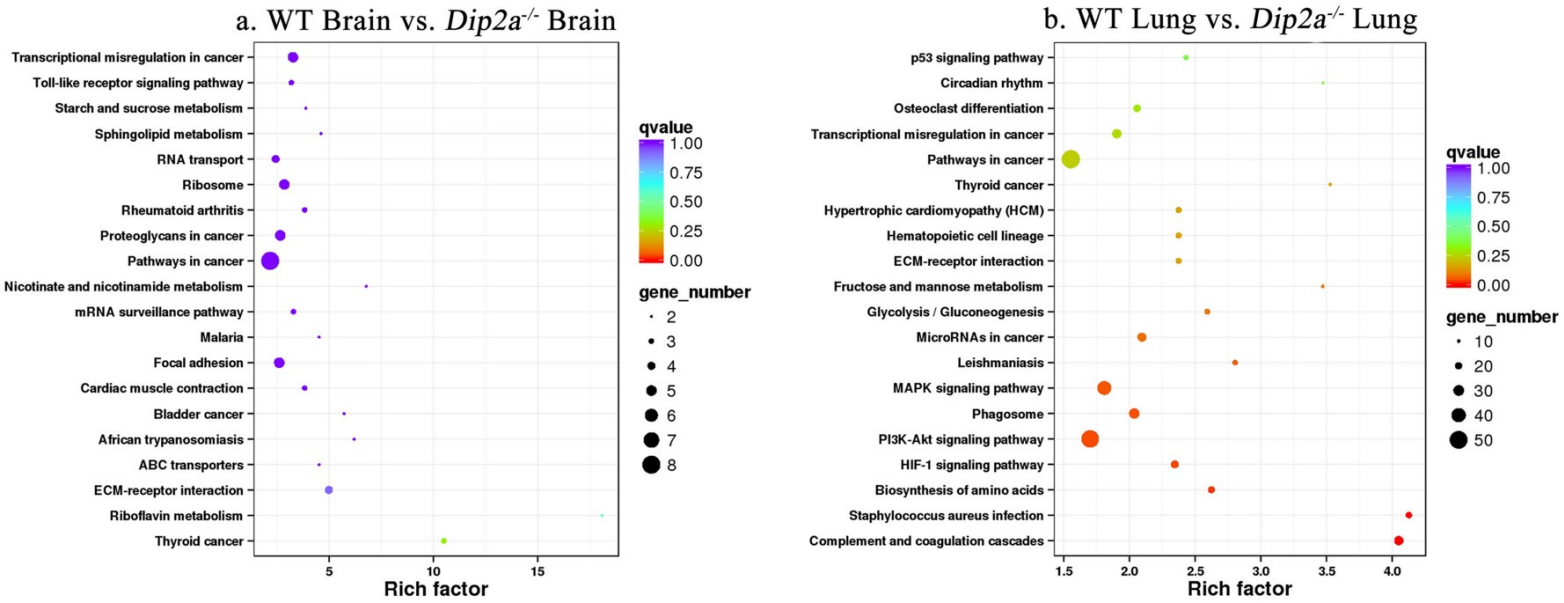
KEGG pathway enrichment scatter plot of DEGs. (a) WT brain vs. *Dip2a*^-/-^ brain and (b) WT lung vs. *Dip2a*^-/-^ lung. Each circle in the figure represents a KEGG pathway. The Y-axis represents name of the pathway and the X-axis indicates Enrichment Factor, indicating the proportion of the annotated genes to all genes in the pathway.

In the process of pathways annotation for *Dip2a* regulated DEGs, 70 DEGs from brain and 625 DEGs from lung were annotated to 112 and 264 pathways respectively in KEGG pathway database (S1 Fig). In order to analyze whether DEGs are over-presented on a pathway, the pathway enrichment analysis was performed (Fig 6). The top 5 enriched pathways in brain with the least significant Q value<0.05 and enrichment factor greater than 2 were ‘ko04610 Complement and coagulation cascades’, ‘ko05150 *Staphylococcus aureus* infection’, ‘ko01230 Biosynthesis of amino acids’, ‘ko04066 HIF-1 signaling pathway’ and ‘ko04151 PI3K-Akt signaling pathway’, whereas in lung, the most enriched pathways with the least Q value<1 and enrichment factor> 2 are ‘ko05216 Thyroid cancer’, ‘ko00740 Riboflavin metabolism’, ‘ECM-receptor interaction’, ‘ko05202 Transcriptional misregulation in cancer’ and ‘ko05200 Pathways in cancer’.

### Transcription factor annotation of DEGs

Zinc finger domain containing transcription factor are the most abundant proteins whose function are extraordinarily diverse and include epithelium development, neo-cortex development, transcription activation, regulation of apoptosis, protein folding and assembly [13–14]. *Dip2a* is thought to be a transcription factor due to its zinc finger motif [2]. To extend these findings, 14 DEGs (9 up & 5 down) from brain and 203 DEGs (163 up & 40 down) from lung were annotated with transcription factor (animal TFDB) database. In both group, the most of up-regulated genes belongs to Zinc finger Cys_2_His_2_-like class group (ZF-C2H2) [124 & 2], Homeobox (5 & 2), High-mobility group (HMG) [4 & 1], Zinc finger and BTB domain-containing protein (ZBTB) [4 & 1], whereas the most of down-regulated genes accounts to transcription factor basic leucine zipper domain (TF-bZIP) [8 & 1], Thyroid hormone receptor [2&1] and Interferon regulatory factor (IRF) [2,1]. Based upon these evidences, our study strongly suggests that DIP2A protein regulate expression of Zinc Finger domain containing proteins during lung and brain development. Transcription factor with the highest fold change (FC>6) from each group is listed in Table 3.

**Table 3:** List of highly differentially expressed Transcription factors (FC>6, FDR<0.001) in WT lung vs. Dip2a^-/-^ lung and WT brain vs. Dip2a^-/-^ brain respectively.

**Gene ID**	**Gene Symbol**	**log2FC**	**TF Family**
ENSMUSG00000090093	*Gm14399*	4.118479	ZF-C2H2
ENSMUSG00000056824	*Zfp663*	3.892387	ZF-C2H2
ENSMUSG00000074867	*Zfp808*	3.841335	ZF-C2H2
ENSMUSG00000078902	*Gm14443*	3.669152	ZF-C2H2
ENSMUSG00000078864	*Gm14322*	3.530807	ZF-C2H2
ENSMUSG00000031079	*Zfp300*	3.363612	ZF-C2H2
ENSMUSG00000074865	*Zfp934*	3.350508	ZF-C2H2
ENSMUSG00000078502	*Gm13212*	3.262812	ZF-C2H2
ENSMUSG00000061371	*Zfp873*	3.252192	ZF-C2H2
ENSMUSG00000090015	*Gm15446*	3.222472	ZF-C2H2
ENSMUSG00000046351	*Zfp322a*	3.212515	ZF-C2H2
ENSMUSG00000055240	*Zfp101*	3.180478	ZF-C2H2
ENSMUSG00000030393	*Zik1*	3.146957	ZF-C2H2
ENSMUSG00000069184	*Zfp72*	3.037894	ZF-C2H2
ENSMUSG00000078899	*Gm4631*	3.009177	ZF-C2H2
ENSMUSG00000028341	*Nr4a3*	-4.48349	NOR
ENSMUSG00000024176	*Sox8*	-3.77856	HMG
ENSMUSG00000024912	*Fosl1*	-3.34232	TF_BZIP
ENSMUSG00000003545	*Fosb*	-3.01894	TF_BZIP
**Gene ID**	**Gene Symbol**	**log2FC**	**TF Family**
ENSMUSG00000001444	*Tbx21*	4.909944	T-BOX
ENSMUSG00000067261	*Foxd3*	4.204621	FORK HEAD
ENSMUSG00000055102	*Zfp819*	3.790954	ZF-C2H2
ENSMUSG00000022479	*Vdr*	-3.50616	THYROID RECEPTOR HORMONE
ENSMUSG00000070031	*Sp140*	-3.024071	SAND
ENSMUSG00000024986	*Hhex*	-3.013431	HOMEOBOX

### DEGs validation by quantitative real-time PCR

**Fig 7:**
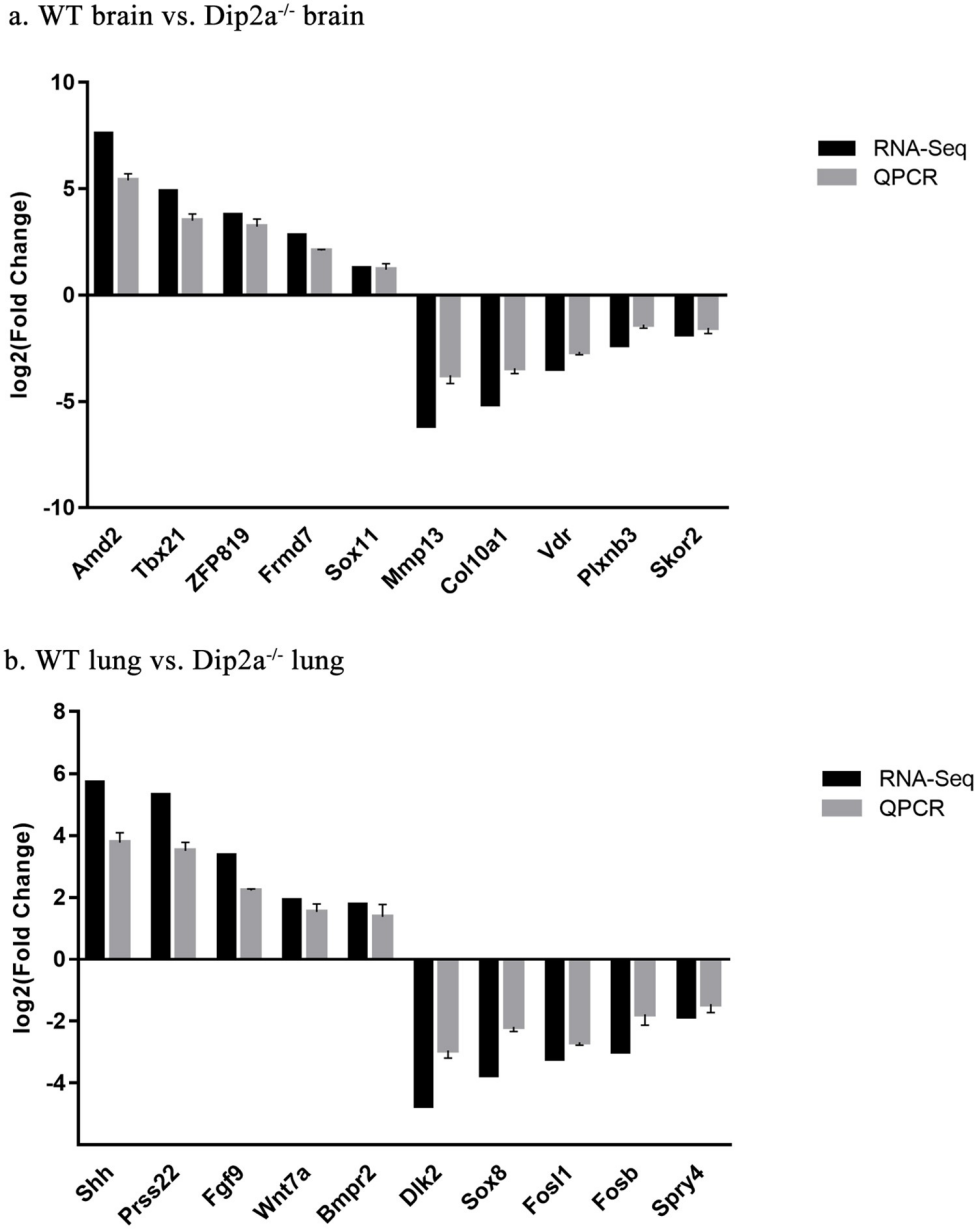
Validation of RNA-Seq results by real-time quantitative PCR (QPCR).

To evaluate validity of RNA-Seq data, five up-regulated DEGs and five-down regulated DEGs from each group were selected for quantitative real-time RT-PCR (qPCR) (Fig 7). The RNA-Seq results of these genes were similar to those obtained by qPCR. These results confirmed the good quality of RNA-Seq results.

### Roles of *Dip2a* in neuronal cell maturation, differentiation and survival

Previous studies have suggested that *Dip2a* is highly expressed in neuronal cells of developing central nervous system such as retinal ganglion cells, Purkinje cell layer and granular cell, and may play important roles in synapse formation and axon guidance [1–4]. In this study, we found 10 genes that are important in neuronal cell maturation and in brain development were differentially expressed in *Dip2a*^-/-^ brain. *Skor2* and *Gpr37l1* genes important in Purkinje cell maturation, differentiation and layer formation were down- regulated [15–16]. Runx1 gene is an important in cell fate specification and axonal projections of dorsal root ganglion neurons and *Erbb3* gene is required in the control of growth and development of Schwann cell [17–18]. These genes were down-regulated. Similarly, *Frmd7* gene which promotes neuronal outgrowth and migration of neural precursor cell was up-regulated [19]. *Fut10* is important in maintenance and differentiation of neuron stem cell and was up-regulated [20]. Extracellular matrix component *Hapln1* gene that plays an important in neo-cortex development and expansion was found over expressed [21]. Transcription factor SRY-box (Sox) family gene *Sox11* is expressed abundantly in all type of embryonic sensory neurons including sensory ganglion and trigeminal ganglion and promotes neuronal maturation was found up-regulated [22]. In addition, transcription factor AP-2 family gene *Tfap2c* important in neural crest induction was under expressed [23]. We also found *Plxnb3* gene was down-regulated. Increasing evidence suggests that Plexin-B3 is axon guidance molecule and promotes synapse formation in rat hippocampal neurons [24]. Hence, these finding strongly supports the role of *Dip2a* in all type of neuronal cell maturation, differentiation and survival.

### Roles of *Dip2a* in lung development

*Dip2a* gene role in lung development has not been symmetrically studied before. In this study, we found significantly altered expression of multiple genes known to participate in lung development. Among them include genes important in epithelial and mesenchyme cell proliferation and differentiation, vasculogenesis, alveologenesis and branching morphogenesis. *Hoxa5*, *Sox11*, *Errfi1* and *Eya1* genes important in embryonic respiratory tract morpogenesis/organogenesis, lung epithelial, mesenchymal and vascular development were up-regulated [25–28]. *Ccbe1* gene is required for development of lymphatic vascular network and was found down-regulated [29]. Similarly, *Lama5* gene needed for proper immune system process was down-regulated [30]. *Rbp4* and *Wnt7a* genes play an important role in alveologenesis were also found under expressed [31–32]. *FGF9* gene is expressed in the pulmonary epithelium and is needed for epithelial branching was over expressed [33]. Pleiotrophin (*Ptn*) gene is involved in fibroblast and epithelial cell communication during fetal lung development was up-regulated [34]. *IGF-1* signaling modulates the development and differentiation of many types of lung cells, including airway basal cells, club cells, alveolar epithelial cells, and fibroblasts was over expressed [35]. In addition, *Dhcr7* gene plays an important role in lung saccular development was also up- regulated [36]. *Crh* gene required for epithelial and mesenchyme cell proliferation was under expressed [37]. *Pdgfra* is known to regulate cell differentiation, proliferation, migration, actin reorganization and apoptosis was under represented [38].

## Conclusion

In this report, four Transcriptome, including WT brain and lung, *Dip2a*^-/-^ brain and lung at embryonic E19.5 were analyzed. On an average 6000 unigenes in each sample were generated with the Illumina Hiseq^™^ 2500 platform. In WT brain vs. *Dip2a*^-/-^ brain comparison, a total of 214 DEGs were detected, including 82 up- and 132 down-regulated genes. These DEGs included genes involved in neuronal cell maturation, differentiation and survival. In WT lung vs. *Dip2a*^-/-^ lung comparison, a total of 1900 DEGs were detected, including 1259 up- and 641 down-regulated genes. These DEGs are important in apoptosis process, lung epithelial development and in morphogenesis. To conclude, we have identified several candidate genes that are regulated by *Dip2a* at E19.5 brain and lung. It would be interesting to further study the biological functions of these genes in brain and lung development.

## Supporting information

S1 Table BLAST analysis of the non-redundant DEGs against six public databases. (TIF)

S1 Fig Annotated diagram of the KEGG pathway of differentially expressed genes; (a) WT lung vs. *Dip2a*^*-/-*^ lung (b) WT brain vs. *Dip2a*^*-/-*^ brain. (TIF)
