# Correction: Transcriptome profiling of mouse brain and lung under Dip2a regulation using RNA-sequencing

**DOI:** 10.1371/journal.pone.0225570

**Published:** 2019-11-14

**Authors:** Rajiv Kumar Sah, Anlan Yang, Fatoumata Binta Bah, Salah Adlat, Ameer Ali Bohio, Zin Mar Oo, Chenhao Wang, May Zun Zaw Myint, Noor Bahadar, Luqing Zhang, Xuechao Feng, Yaowu Zheng

The second author's name is spelled incorrectly. The correct name is: Anlan Yang. The correct citation is: Sah RK, Yang A, Bah FB, Adlat S, Bohio AA, Oo ZM, et al. (2019) Transcriptome profiling of mouse brain and lung under Dip2a regulation using RNA-sequencing. PLoS ONE 14(7): e0213702. https://doi.org/10.1371/journal.pone.0213702
