# Supplementary figures and images for: Transcriptome profiling of mouse brain and lung under Dip2a regulation using RNA-sequencing
Source: PLoS One. 2019 Jul 10;14(7):e0213702. doi: 10.1371/journal.pone.0213702 (PMC6619597; doi:10.1371/journal.pone.0213702)

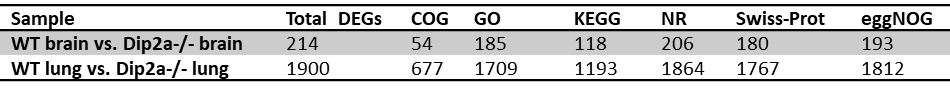

Supplement: S1 Table — (TIF) [file pone.0213702.s001.tif]

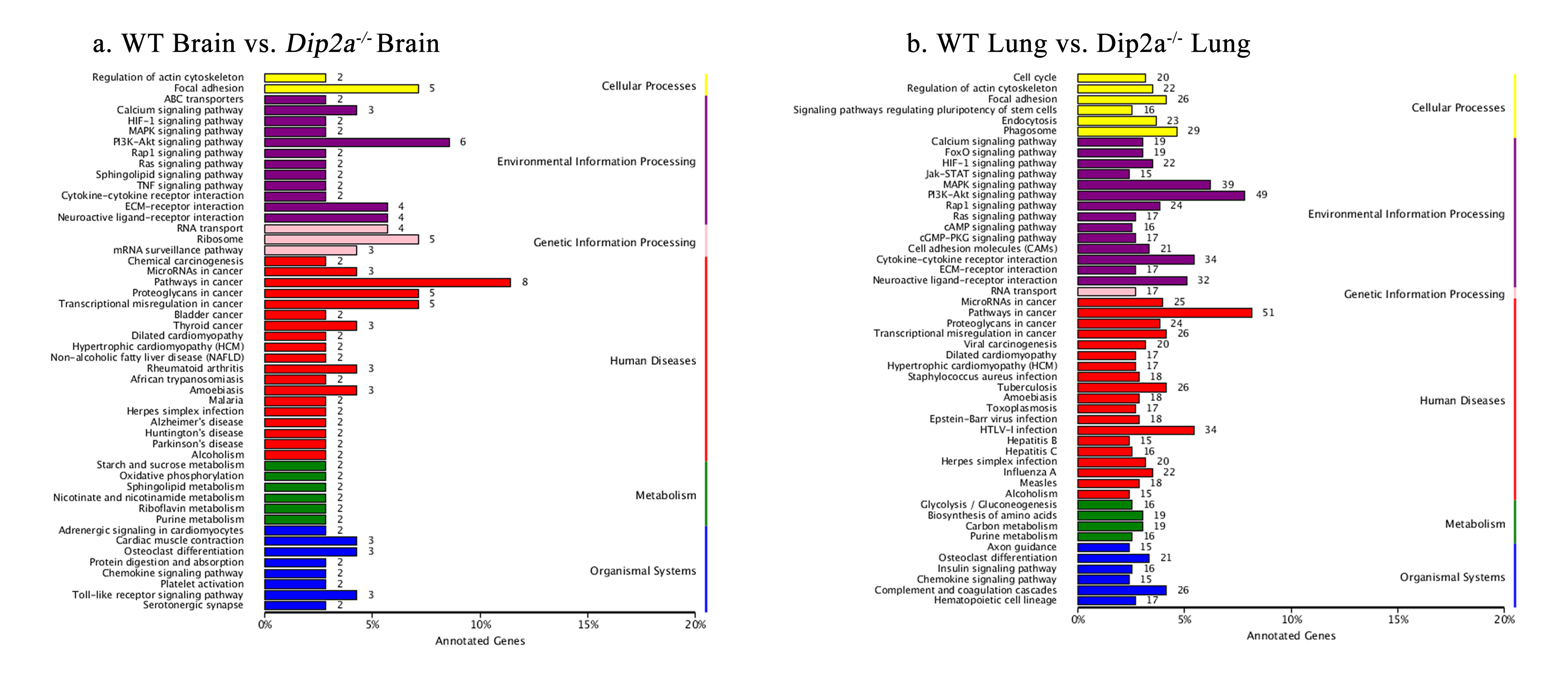

Supplement: S1 Fig — (TIF) [file pone.0213702.s002.tif]
